# Investigation of *Salmonella enteritidis* Growth under Varying Temperature Conditions in Liquid Whole Egg: Proposals for Smart Management Technology for Safe Refrigerated Storage

**DOI:** 10.3390/foods13193106

**Published:** 2024-09-28

**Authors:** Seung-Hee Baek, Chang-Geun Lim, Jung-Il Park, Yeon-Beom Seo, In-Sik Nam

**Affiliations:** 1 Research Center for Environmentally Friendly and Quality Livestock Production Technology, Hankyong National University, Anseong-si 17579, Gyeonggi-do, Republic of Korea; shbaek@hknu.ac.kr; 2 School of Animal Life Convergence Science, Hankyong National University, Anseong-si 17579, Gyeonggi-do, Republic of Korea; dlackdrms921@naver.com (C.-G.L.);; 3 Korea Agency of HACCP Accreditation and Services, Cheongju-si 28165, Chungcheongbuk-do, Republic of Korea; 4 Institute of Applied Humanimal Science, Hankyong National University, Anseong-si 15759, Gyeonggi-do, Republic of Korea

**Keywords:** *Salmonella enteritidis*, non-isothermal conditions, predictive growth modeling, temperature control, food safety

## Abstract

This study investigates the growth characteristics of *Salmonella enteritidis* (*S. enteritidis*) in liquid whole egg under both isothermal and non-isothermal storage conditions to understand the risks associated with inadequate temperature management in the egg industry. Using controlled laboratory simulations, liquid whole egg samples inoculated with *S. enteritidis* were stored under various isothermal (5, 15, 25, 35, and 45 °C) and non-isothermal conditions (5–10, 15–20, 25–30, 35–40, and 45–50 °C). The growth behavior of the *S. enteritidis* was analyzed using a two-step predictive modeling approach. First, growth kinetic parameters were estimated using a primary model, and then the effects of temperature on the estimated specific growth rate and lag time were described using a secondary model. Independent growth data under both isothermal and non-isothermal conditions were used to evaluate the models. The results showed that *S. enteritidis* exhibits different growth characteristics depending on temperature conditions, emphasizing the need for strict temperature control to prevent foodborne illnesses. To address this, a predictive growth model tailored for non-isothermal conditions was developed and validated using experimental data, demonstrating its reliability in predicting *S. enteritidis* behavior under dynamic temperature scenarios. Additionally, temperature management technologies were proposed and tested to improve food safety during refrigerated storage. This study provides a scientific basis for improving food safety protocols in the egg industry, thereby protecting public health and maintaining consumer confidence amid temperature fluctuations.

## 1. Introduction

*Salmonella* is a leading cause of foodborne illness worldwide, with annual cases of foodborne salmonellosis estimated conservatively at 80.3 million, though some estimates suggest figures as high as 1.3 billion [1]. In 2017 in the United States, according to the investigation of foodborne disease outbreaks and outbreak-associated illnesses, there were a total of 271 outbreaks caused by bacteria, with *Salmonella* being the most common, responsible for 122 outbreaks and 3061 illnesses. [2]. In the food category of land animals, there were a total of 83 outbreaks, with chicken accounting for 23 outbreaks, beef for 19 outbreaks, and dairy for 14 outbreaks. Among these, chicken was responsible for 487 illnesses. In 2121 in the EU, there were a total of 60,050 confirmed human cases of *Salmonella*, with 773 resulting from foodborne outbreaks, leading to a total of 6755 illnesses. Specifically, there were 39 foodborne outbreaks associated with eggs and egg products. The top five *Salmonella* serovars responsible for human infections were *Salmonella enteritidis* (54.6%), *Salmonella typhimurium* (11.4%), monophasic *Salmonella typhimurium* (8.8%), *Salmonella infantis* (2.0%), and *Salmonella derby* (0.93%) [3]. 

*Salmonella* can be transmitted through both vertical and horizontal transmission among poultry, and this can lead to human infection. Vertical transmission occurs when infected parent poultry pass the bacteria to their offspring through eggs, while horizontal transmission spreads the bacteria among poultry through contaminated feces, feed, or water. Additionally, *Salmonella* can be transmitted to humans through the consumption of contaminated eggs or chicken, as well as through cross-contamination in slaughterhouses [4]. *Salmonella* foodborne disease is closely linked to the presence of *S*. *enteritidis*, indicating that poultry products are major sources of infection. Eggs are one of the most widely consumed foods globally, and are valued for their nutritional content and culinary versatility. However, they also pose a significant risk as a vector for *Salmonella*. Particularly liquid egg products, which are widely used as ingredients in various food products, can become contaminated either by using *Salmonella*-infected eggs or through cross-contamination from improperly sanitized equipment [5,6]. The potential for *Salmonella* contamination in eggs has led to a heightened focus on food safety measures throughout the poultry industry. 

In South Korea, Hazard Analysis and Critical Control Point (HACCP) has been rigorously implemented in food and livestock manufacturing facilities to address these concerns [7]. HACCP is a systematic approach that identifies, evaluates, and controls hazards that are significant for food safety. Within this framework, refrigerated storage processes are designated as critical control points (CCP) due to the role temperature plays in inhibiting microbial growth. Regular monitoring and strict temperature control are mandated to minimize the risk of *Salmonella* proliferation [8]. Despite these stringent controls, the effectiveness of temperature management is often challenged by operational practices and environmental factors. For instance, during the storage and handling of products, frequent door openings for product loading and unloading can result in significant temperature fluctuations, even in refrigerated environments. Equipment malfunctions and improper handling further exacerbate the risk of temperature deviations, leading to conditions that may allow *Salmonella* to survive and multiply. The persistence of this pathogen under suboptimal conditions underscores the importance of robust and responsive temperature control measures.

This issue is not merely a theoretical concern. According to several studies, even slight deviations from optimal storage temperatures can lead to a significant increase in the growth of *Salmonella* spp. in eggs [9,10,11]. Refrigeration alone is not fail-safe and, if temperatures are not maintained consistently within the safe range, *Salmonella* can proliferate, leading to contaminated products reaching consumers. This situation is particularly concerning given the widespread consumption of eggs and the potential for outbreaks of foodborne illness. To address these issues, it is crucial to explore the growth characteristics of *Salmonella* under varying temperature conditions. Understanding how *Salmonella* behaves under both isothermal (constant temperature) and non-isothermal (fluctuating temperature) conditions provides valuable insights into its survival and proliferation dynamics [12,13,14]. Such knowledge is essential for developing more effective temperature management strategies that can be implemented in practical environments, where perfect conditions are rarely maintained.

The main objective of this study is to investigate the growth characteristics of *Salmonella* in liquid whole egg under controlled laboratory conditions that simulate both isothermal and non-isothermal storage scenarios. Inadequate temperature management can have a negative impact on the shelf life, quality, and safety of these products. Therefore, our study aims to analyze pathogen growth under various conditions to identify key factors influencing *Salmonella* proliferation and to quantify the risks associated with temperature fluctuations. To achieve this, it is necessary to develop a non-isothermal model that can explain the impact of temperature changes on microbial growth and to establish and validate temperature management practices that can minimize the risk of *Salmonella* contamination during refrigerated storage.

The findings will not only contribute to a better understanding of *Salmonella* behavior in eggs but will also provide a scientific basis for improving food safety protocols in the egg industry. By enhancing temperature control measures and ensuring that they are rigorously applied, it is possible to reduce the incidence of *Salmonella*-related foodborne illness, protect public health and maintain consumer confidence in egg products.

## 2. Materials and Methods

### 2.1. Sample Preparation

The liquid whole eggs used in this study were commercially available products purchased directly. The samples were stored in a refrigerator at 5 °C until they were used in the experiment. To verify the presence of *Salmonella* spp. contamination in the samples, a qualitative test was conducted according to the microbial testing method described in the Korean Food Code [15]. A 25 mL sample was added to 225 mL of Buffered Peptone Water (BPW, MB Cell, Seoul, Republic of Korea) and incubated at 36 ± 1 °C for 24 h. From the cultured BPW, 1 mL and 0.1 mL were taken and added to 10 mL of Tetrathionate broth (MB Cell, Seoul, Republic of Korea) and 10 mL of Rappaport-Vassiliadis broth (MB Cell, Seoul, Republic of Korea), respectively, followed by incubation at 36 ± 1 °C and 41.5 ± 1 °C for 24 h, respectively. The incubated broths were then streaked onto Xylose Lysine Deoxycholate agar (XLD, MB Cell, Seoul, Republic of Korea) and Brilliant Green Sulfa agar (MB Cell, Seoul, Republic of Korea), and incubated at 36 ± 1 °C for 24 h, respectively. The qualitative test for *Salmonella* spp. on the whole egg liquid purchased for this experiment was determined to be negative.

### 2.2. Inoculation and Enumeration

*Salmonella enteritidis* (*S. enteritidis,* NCCP 16947) was obtained from the National Culture Collection for Pathogens (NCCP) in Korea and cryopreserved at −70 °C. The cryopreserved strains were inoculated into Tryptic Soy Broth (BD Difco, Sparks, MD, USA) and incubated at 37 °C for 24 h, followed by a second incubation under the same conditions. The cultures were then streaked onto Tryptic Soy Agar (BD Difco, Sparks, MD, USA) and incubated at 37 °C for 24 h. 

An aliquot (10 mL) of the liquid whole egg was put into a 50 mL tube and inoculated with the *S. enteritidis* at an initial concentration of 5 log CFU/g. Inoculated liquid whole eggs were stored at 5, 15, 25, 35, and 45 °C (*n* = 10–11 samples per temperature). *S. enteritidis* growth data were collected under both isothermal and non-isothermal conditions at these temperatures. For the non-isothermal conditions, a temperature range of +5 °C (5–10 °C, 15–20 °C, 25–30 °C, 35–40, and 45–50 °C) was applied and four cycles were completed every 24 h, following a modified method based on Costa et al. [16]. The collected growth data were then used to calculate performance measures for the predictive model (described below).

When the storage time ended, each sample was removed from the incubator, homogenized, and then subjected to serial dilutions before being streaked onto XLD agar. Each medium was incubated in a 37 °C incubator for 24 h, and the number of cells was counted to calculate log CFU/mL.

### 2.3. Modeling

#### 2.3.1. Primary Model

The primary growth predictive model (Equation (1)) is based on the Baranyi function equation proposed by Baranyi and Robert [17]. Parameters, such as lag time (LT; h) and maximum specific growth rate (*μ_max_*; log CFU/g/h), were calculated by fitting the growth data to a predictive model using DMFit software, a tool provided by ComBase (Institute of Food Research, Norwich, UK). The Baranyi model was as follows:

(1) $$y(t)=y_{0}+\mu_{\max}\;F(t)-ln\left(1+\frac{{exp}^{\;\mu_{\max\;}F(t)}-1}{{exp}^{\left(y_{\max\;}-y_{0}\right)}}\right)\;F\left(t\right)=t+\frac{1}{v}ln\left(e^{-vt}+e^{-h_{0}}-e^{\left(-vt-h_{0}\right)}\right),$$

In the equation, *y*(*t*) is the cell density (ln CFU/g) at time *t*, y_0_ is the initial cell density (ln CFU/g), *y_max_* is the maximum cell density (ln CFU/g), *µ_max_* is the specific growth rate (ln CFU/g/h), *v* is the rate of increase in the limiting substrate (assumed to be equal to *µ_max_*), and *h*_0_ is calculated by multiplying the specific growth rate by the lag time.

#### 2.3.2. Secondary Model

The secondary model describes the effects of temperature on the parameters of the primary model. The modified Ratkowsky [18] (Equation (2)) and polynomial [19] models (Equation (4)) were used to analyze the effects of temperature on the specific growth rate (*µ_max_*) and lag time (*LT*), respectively, calculated from the primary models using Minitab 19 (Minitab LLC, State College, PA, USA) and GraphPad Prism 9.0 (GraphPad Software, San Diego, CA, USA USA).

(2) $$\mu_{\max}=a(T-T_{\min}{)}^{2}\;\left(1-exp\left(b\left(T-T_{\max}\right)\right)\right),$$

where *T_min_* and *T_max_* represent the theoretical minimum and maximum temperatures beyond which growth does not take place, with a and b as regression constants. For *Salmonella*, temperatures between 5 and 10 °C and 42 and 50 °C are typically used as starting values [20]. In this study, 5 °C and 45 °C, where growth data were collected, were designated as the starting values for *T_min_* and *T_max_*. The parameter *a* was determined by calculating the slope of $\sqrt{\mu_{\max}}$ plotted against temperature until the maximum growth rate was reached (Equation (3)):

(3) $$\sqrt{\mu_{\max}}=b(T-T_{\min}),$$

The *µ_max_* values were estimated from each primary model at different isothermal and non-isothermal temperatures. The starting value for *b* was set to 1.

(4) $$LT\;and\;\mu_{\max}=b_{0}+\left(b_{1}\times T\right)+(b_{2}\times T^{2}),$$

where *b*_0_, *b*_1_, and *b*_2_ are regression constants and *T* represents temperature.

#### 2.3.3. Validation of Growth Predictive Model

To validate the performance of the primary and secondary models, five statistical indices were used: bias factor (*B_ƒ_*), accuracy factor (*A_ƒ_*), root mean square error (*RMSE*), Akaike information criterion (*AIC*), and Bayesian information criterion (*BIC*), as represented by Equations (5)–(9) [20].

(5) $$B_{f}=10\frac{\sum log\left(\frac{Pred}{Obs}\right)}{n},$$

(6) $$A_{f}=10\frac{\sum |log\left(\frac{Pred}{Obs}\right)|}{n},$$

(7) $$RMSE=\sqrt{\sum {\left(\frac{Obs-Pred}{n}\right)}^{2}},$$

The variables include (*Obs*) for observed values, (*Pred*) for predicted values, and n representing the number of observations.

(8) $$AIC=n\;ln\left(\frac{SSE}{n}\right)+2p,$$

(9) $$BIC=n\;ln\left(\frac{SSE}{n}\right)+2\left(p+2\right)q-{2q}^{2},$$

In Equations (8) and (9), *n* is the number of observations or data points, SSE is the sum of squares of errors in the model, and *p* is the number of parameters in the model. In Equation (9), *q* is calculated as *nσ*^2^/SSE, where *σ*^2^ represents the estimate of error variance from fitting the full model.

A model is considered suitable if the bias factor (*B_ƒ_*) is between 0.7 and 1.15, the accuracy factor (*A_ƒ_*) is close to 1, and the values of RMSE, AIC, and BIC are low, indicating accurate predictions for the growth of *S. enteritidis*.

## 3. Results

### 3.1. Growth Characteristics

**Figure 1:**
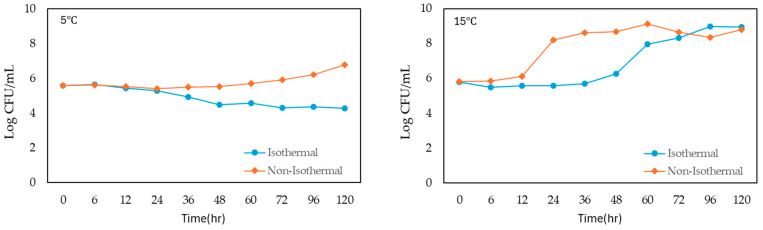

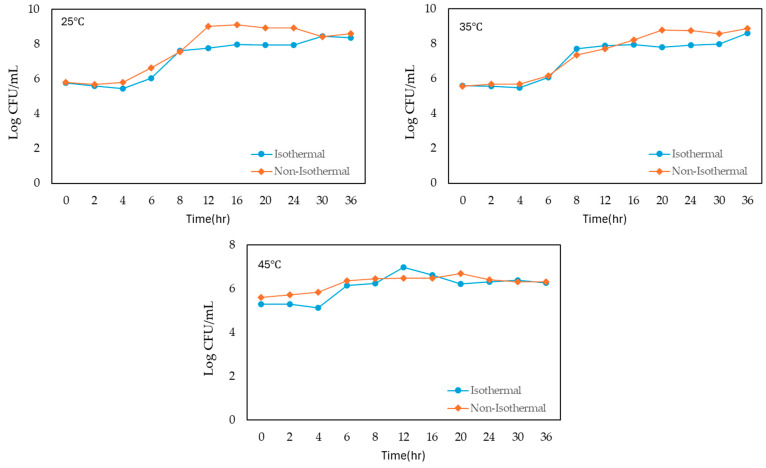
Growth of *S. enteritidis* on liquid whole egg at 5 °C, 15 °C, 25 °C, 35 °C and 45 °C.

The growth of *S. enteritidis* in liquid whole egg was monitored under both isothermal and non-isothermal conditions across a range of temperatures from 5 °C to 45 °C, with incubation times extending up to 36–120 h (Figure 1). The initial bacterial concentration was approximately 5 log CFU/mL in all conditions.

Under isothermal conditions, *S. enteritidis* showed a slight initial increase in population at the lowest temperature of 5 °C and peaked at 5.655 log CFU/mL after 6 h. However, the bacterial count then decreased steadily over time, reaching a final concentration of 4.273 log CFU/mL at 120 h. At 15 °C, the growth was more gradual, and the population reached 5.569 log CFU/mL after 24 h and continuing to increase to 8.937 log CFU/mL at 120 h. At 25 °C, bacterial growth was more rapid, and the population reached 6.033 log CFU/mL by 6 h and peaking at 8.463 log CFU/mL by 30 h, followed by a slight decline. At 35 °C, *S. enteritidis* demonstrated the most rapid growth, and the population surged to 7.717 log CFU/mL within 8 h and continued to 8.615 log CFU/mL by 36 h.

Under non-isothermal conditions, *S. enteritidis* exhibited various growth patterns. At 5 °C, the population showed a similar initial increase but remained relatively stable, with a final count of 6.767 log CFU/mL at 120 h. At 15 °C, growth was more pronounced, with the population rapidly increasing to 8.627 log CFU/mL by 36 h and reaching 8.791 log CFU/mL at 120 h. At 25 °C, the population reached 8.923 log CFU/mL at 20 h, peaking at 8.614 log CFU/mL by 36 h. At 35 °C, bacterial growth was the fastest, with the population reaching 8.882 log CFU/mL by 36 h. However, at 45 °C, the population increased gradually, reaching 6.321 log CFU/mL by 36 h from the beginning of storage.

### 3.2. Primary Modeling

The growth kinetics of *S. enteritidis* in liquid whole egg were evaluated under both isothermal and non-isothermal conditions across a range of temperatures. The results were fitted to the Baranyi model for all data obtained under isothermal and non-isothermal conditions, as summarized in Table 1. At 15 °C, the isothermal condition exhibited a lag time of 15.932±1.880 h with a specific growth rate of 0.041±0.001 log CFU/g, while the non-isothermal condition had a reduced lag time of 11.472±1.369 h and a much higher specific growth rate of 0.201±0.038 log CFU/g. At higher temperatures (25 °C and 35 °C), non-isothermal conditions consistently resulted in shorter lag times and lower specific growth rates than isothermal conditions, highlighting the impact of fluctuating temperatures on growth kinetics. The B*_f_* and accuracy factor A*_f_* were close to 1 for all models. This indicated that the models provided reliable predictions of *S. enteritidis* growth. These findings show that the growth of *S. enteritidis* in non-isothermal environments affects the lag time and growth rate differently compared to stable temperatures.

### 3.3. Secondary Modeling

Secondary models were developed to describe the lag time and specific growth rate of *S. enteritidis* in liquid whole egg under both isothermal and non-isothermal conditions, as shown in Table 2. Using parameters derived from primary models, polynomial and modified Ratkowsky models were constructed to characterize the growth kinetics. The estimated T_min_ and T_max_ for *S. enteritidis* in liquid whole egg were 7.9 °C and 45.8 °C under isothermal conditions, and 2.7 °C and 49.5 °C under non-isothermal conditions, respectively. The performance of these models was evaluated using AIC, BIC, and RMSE values. Under isothermal conditions, the modified Ratkowsky model showed a more accurate fit for μ_max_ compared to the polynomial model, with low AIC, BIC, and RMSE values. However, under non-isothermal conditions, the polynomial model provided the best fit to describe the growth rate and had the lowest values. Both the polynomial and the modified Ratkowsky models proved to be more effective in capturing the complex dynamics of microbial growth for μ_max_ under both isothermal and non-isothermal temperature conditions. This suggests the robustness and suitability of the models for predicting microbial behavior in various environmental scenarios. 

### 3.4. Validation of the Models

**Figure 2:**
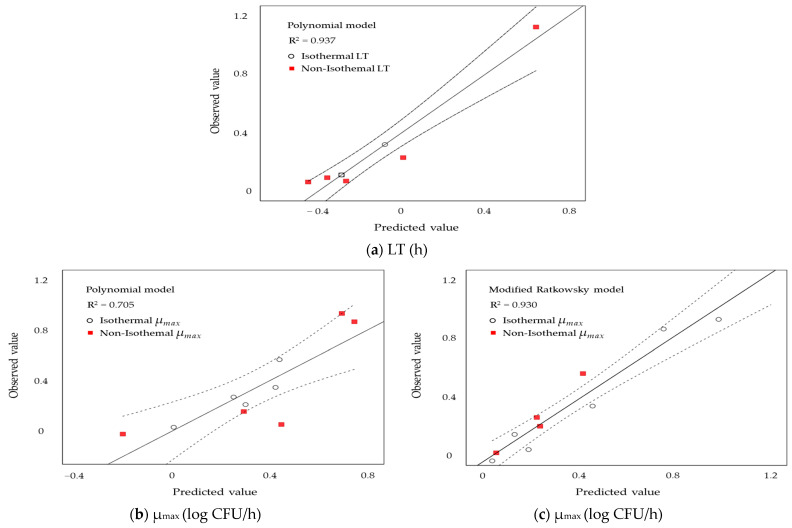
Comparisons of observed and predicted values of *S. enteritidis* growth on liquid whole egg, based on lag time (LT) values derived from the polynomial models (**a**) and specific growth rate (µ_max_) values derived from the polynomial (**b**) and modified Ratkowsky (**c**) models, are shown.

As shown in Figure 2, The observed LT values (a) closely matched the predicted values, demonstrating a relatively high goodness-of-fit (R^2^ = 0.937) for the predictive models. The R^2^ values for the observed μ_max_ from the polynomial and Modified Ratkowsky models were 0.705 and 0.930, respectively, indicating that the Modified Ratkowsky model is more appropriate. Comparing the predicted values with the observed values suggested that linear regression could provide predictions closer to the experimental data.

**Figure 3:**
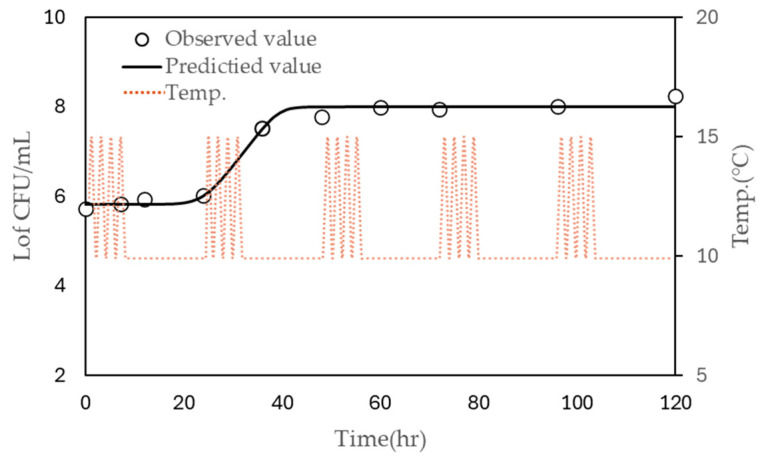
Observed (symbols) and predicted (continuous line) growth of *S. enteritidis.* in liquid whole egg stored at 10 °C under non-isothermal conditions (dashed line), with four cycles every 24 h.

The models were further validated using growth data obtained from inoculated liquid whole egg stored under non-isothermal conditions, where the temperature fluctuated between 10 °C and 15 °C over a 24-h period with four cycles of temperature change (Figure 3). The observed LT and μ_max_ were 31.776 h and 0.076, respectively, while the predicted LT was 34.498 h. The predicted µmax values were 0.163 using the polynomial model and 0.142 using the modified Ratkowsky model. These results indicate that the predictions of the model are reasonably close to the observed values and demonstrate the model’s reliability for predicting microbial growth under fluctuating temperature conditions.

## 4. Discussion

Several studies have demonstrated the advantages of the Baranyi model in specific situations. Baranyi and Roberts [17] highlighted the model’s superior performance in describing bacterial growth under fluctuating environmental conditions. Similarly, Kim et al. [14] investigated the growth characteristics of *Salmonella* in unpasteurized liquid eggs, Lin et al. [21] studied the growth of *Salmonella* spp. in liquid egg products, and Costa et al. [16] examined bacterial growth under non-isothermal conditions. Ye et al. [22] reported that primary models, such as the Baranyi, Modified Gompertz, and logistic models, can effectively model pathogen growth. Juneja et al. [13] evaluated logistic, Modified Gompertz, Baranyi, and Huang models to describe the growth of *Salmonella* spp. under nine different isothermal conditions. They found that all models fit the data well, with no significant differences in performance between the models. While empirical models (Modified Gompertz and logistic models) improve accuracy with complete growth curves, mechanistic models (Baranyi and Huang models) can fit data even without a stationary phase. In this study, the Baranyi model was applied as the primary model and demonstrated statistical goodness-of-fit by exhibiting validation factors, such as B*_f_*, A*_f_*, RMSE, AIC, and BIC, across all temperature conditions. These results confirm the suitability of the Baranyi model for modeling the growth of *S. enteritidis* under both isothermal and non-isothermal conditions. Ultimately, the use of this model provided more accurate and reliable predictions of *S. enteritidis* growth, enhancing our understanding of microbial dynamics in liquid whole egg products across various temperature conditions.

Huang [23] used a secondary model to estimate the maximum growth rate (µ_max_) of *S. enteritidis* in pasteurized liquid egg white, reporting values of 0.081 at 10 °C and 0.921 at 37 °C. These estimates are similar to the results of our study, where the µ_max_ was found to be 0.086 at 10 °C and 1.058 at 37 °C. The modified Ratkowsky model is widely applied in studies of *Salmonella* growth [24,25]. The temperature range for *Salmonella* growth is known to be between 5 °C and 45 °C. In our study, the predicted temperature range for *S. enteritidis* was 7.9 °C to 45.9 °C under isothermal conditions and 2.8 °C to 45.9 °C under non-isothermal conditions, aligning with these known values. The consistent performance of the secondary model under both isothermal and non-isothermal conditions suggests that it is a reliable model for explaining *Salmonella* growth.

Lin et al. [21] developed a model to predict Salmonella growth in egg products under both static and fluctuating temperature conditions, confirming its ability to assess the risk of Salmonella growth during temperature abuse in liquid egg processing. Similarly, Singh et al. [26] found that Salmonella grows more rapidly under non-isothermal conditions, as fluctuating temperatures allow the bacteria to stay within the optimal growth range for extended periods. When comparing the model developed by Gumudavelli et al. [24] for egg yolk and the result of our study, it was observed that *Salmonella* spp. growth was under-predicted at low temperatures. These findings align with those of Lin et al. [21] and are likely to be due to differences in food pH, composition, and microorganism types (*Salmonella* spp. vs. *S. Enteritidis*). In our study, *S. enteritidis* exhibited faster growth rates and shorter lag times at 5 °C and 15 °C under non-isothermal conditions, reaching higher bacterial counts more quickly. This emphasizes the importance of maintaining consistent temperature control during refrigeration to reduce the risk of *S. enteritidis* contamination and preserve the quality of liquid whole egg products. We also confirmed that the observed growth rate and lag time under non-isothermal conditions (10 °C, fluctuating between 10 °C and 15 °C) closely matched the values predicted from the model. These results suggest that our study can be useful for predicting *Salmonella* growth under dynamic conditions and for setting safety standards in HACCP plans.

**Figure 4:**
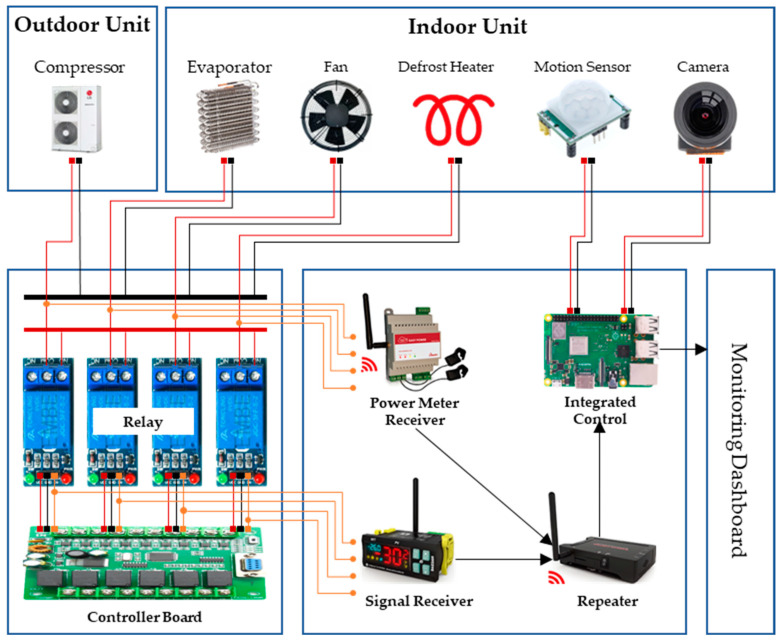
Integrated Sensor Concept for Refrigerated Units.

Through our study, we observed how foodborne pathogens like *Salmonella* grow under non-isothermal conditions and confirmed the importance of strict temperature control in maintaining food quality and safety. In South Korea, HACCP systems are rigorously applied across food and livestock manufacturing facilities, with refrigerated storage processes designated as CCP that are regularly monitored. However, the limitations of manual temperature monitoring can lead to delayed responses to temperature deviations, potentially compromising product safety and quality. To address this, real-time monitoring and precise control are essential, and Internet of Things (IoT) technology makes this possible. IoT-integrated solutions enable continuous tracking and management across the entire process, significantly improving operational efficiency and the ability to maintain safety standards through rapid problem detection and resolution [27,28]. We have developed an integrated sensor system that monitors factors such as door openings and equipment malfunctions, which can lead to temperature deviations, in real-time (Table 3). This system is equipped with temperature sensors, power detection sensors, door opening sensors, and motion sensors, enabling comprehensive environmental monitoring and immediate detection and response to potential issues (Figure 4). As abnormal temperature fluctuations during refrigeration can lead to the proliferation of foodborne pathogens, the system utilizes IoT technology to continuously monitor temperature changes in real time and quickly resolve any issues, ensuring the quality and safety of stored products. This integrated approach significantly enhances the safety and efficiency of food manufacturing processes and allows for proactive management of potential risks.

## 5. Conclusions

In this study, the growth kinetics of *S. enteritidis* in liquid whole egg were investigated under both isothermal and non-isothermal conditions across a temperature range of 5 °C to 45 °C. The Baranyi model was successfully applied as the primary model to describe bacterial growth kinetics, and the results demonstrated that the bias factor (B*_f_*) and accuracy factor (A*_f_*) were close to 1, with low RMSE, AIC, and BIC values, providing reliable predictions. Secondary models, including the polynomial and modified Ratkowsky models, were used to further analyze specific growth rate and lag time across the temperature range. Both models effectively captured the complex dynamics of microbial growth, providing accurate fits. In the validation of the models using experimental data under non-isothermal storage at 10 °C, the predicted specific growth rate and lag time values were reasonably close to the observed values, demonstrating the robustness and suitability of the models for predicting microbial behavior under various temperature conditions.

Overall, the findings confirm that both the primary and secondary models are effective tools for predicting the growth of *S. enteritidis* in liquid whole egg, particularly under non-isothermal conditions, and emphasize the importance of temperature management to ensure food safety. These models can contribute to improving HACCP plans and food safety management systems. Furthermore, by utilizing IoT technology, the system can continuously monitor temperature fluctuations and swiftly address issues, ensuring consistent application of safety standards, while maintaining optimal storage conditions to preserve product safety and quality.

## Figures and Tables

**Table 1:** Parametric values of the primary models of *S. enteritidis* growth on liquid whole egg.

Temp. (°C)	Sample	LT	µ_max_	*B_f_*	*A_f_*	RMSE	AIC	BIC
5	Isothermal	NO	−0.036 ± 0.014	1.000	1.019	0.089	−14.510	−2.246
	Non-isothermal	56.095 ± 2.999	0.019 ± 0.002	1.000	1.014	0.079	−15.483	−2.538
15	Isothermal	15.932 ± 1.880	0.041 ± 0.001	1.005	1.056	0.388	0.852	2.363
	Non-isothermal	11.472 ± 1.369	0.201 ± 0.038	1.001	1.015	0.127	11.600	5.588
25	Isothermal	5.548 ± 0.165	0.864 ± 0.065	1.012	1.030	0.222	2.441	3.860
	Non-isothermal	4.595 ± 0.295	0.561 ± 0.068	1.011	1.027	0.206	10.808	6.142
35	Isothermal	5.538 ± 0.154	0.930 ± 0.193	1.011	1.029	0.207	3.357	4.109
	Non-isothermal	3.140 ± 1.776	0.339 ± 0.218	1.009	1.028	0.198	2.095	3.765
45	Isothermal	3.453 ± 0.000	0.145 ± 0.031	1.011	1.038	0.227	−3.391	2.269
	Non-isothermal	3.459 ± 0.125	0.261 ± 0.065	0.998	1.022	0.137	−4.202	2.048

LT, lag time (h). µ_max_, specific growth rate (log CFU/g). B_f_, bias factor. A_f_, accuracy factor. RMSE, root mean square error. AIC, Akaike’s Information Criterion. BIC, Bayesian Information Criterion. NO, no observation.

**Table 2:** Secondary models developed for lag time (LT) and specific growth rate (*µ_max_*) of *S. Enteritidis* on liquid whole egg and validation factors for evaluating model performance.

Sample	Secondary Model	Equation	AIC	BIC	RMSE
Isothermal	Polynomial model	LT = 50.97 − 3.1114T + 0.05189T^2^	15.93	5.90	0.004
	Polynomial model	*µ_max_* = −0.6781 + 0.1012T − 0.00177^2^	−30.57	−0.39	0.216
	Ratkowsky Model	*µ_max_* = 0.8957(T − 7.883)^2^(1 − exp (0.0001384(T − 45.79)))	−51.31	−5.47	0.081
Non- isothermal	Polynomial model	LT = 73.09 − 4.54T + 0.06808T^2^	63.07	18.34	4.808
	Polynomial model	*µ_max_* = −0.2133 + 0.04552T − 0.0007862T^2^	−52.58	−4.793	0.068
	Ratkowsky Model	*µ_max_* = 0.4843(T − 2.686)^2^(1 − exp (0.00004614(T − 49.47)))	−46.39	−4.488	0.075

LT, lag time (h). *µ_max_*, specific growth rate (log CFU/g/h). AIC, Akaike’s Information Criterion. BIC, Bayesian Information Criterion. RMSE, root mean square error.

**Table 3:** Refrigeration storage integrated sensor implementation technology.

Category	Sensor Applied	Technology Used	Remarks
Implementation Function	Temperature Sensor	Temperature detection	Sensors are individually installed for various management factors in the workplace and are managed by an integrated control panel In case of deviation from the threshold, automatic exception handling is carried out based on sensor detection results
	Defrost Detection Temperature Sensor	Detection of defrost operation status	
	Power Detection Sensor	Detects equipment operation by monitoring changes in power consumption	
	Controller Signal Detection Sensor	Simple detection of door opening during loading/unloading (interlinked with motion sensor)	
	Door Opening Detection Sensor	Detects whether a worker is present during operation by recognizing the worker with motion detection	

## Data Availability

The original contributions presented in the study are included in the article, further inquiries can be directed to the corresponding author.
